# Targeting LMTK3 in ovarian cancer: A dual role in prognosis and therapy

**DOI:** 10.1016/j.omton.2024.200902

**Published:** 2024-11-21

**Authors:** Chrysa Filippopoulou, Georgios Giamas

**Affiliations:** 1 International Oncology Institute, The First Affiliated Hospital of Zhejiang Chinese Medical University, Hangzhou 310053, China; 2 Department of Biochemistry and Biomedicine, School of Life Sciences, University of Sussex, JMS Building, Falmer, Brighton BN1 9QG, UK

Ovarian cancer (OC) remains one of the most lethal gynecological cancers, with limited treatment options, particularly for recurrent disease. To combat this, recent research has focused on identifying molecular drivers that could serve as novel therapeutic targets. In this context, Saed et al. present compelling evidence positioning lemur tail kinase 3 (LMTK3) as both a prognostic biomarker and a prospective novel therapeutic target in OC.^1^ Their study revealed that LMTK3 is expressed in >98% of early-stage OC tumor tissues (*n* = 204). The researchers discovered a correlation between the subcellular localization of LMTK3 in ovarian tumor cells and the clinical outcome of patients with OC. Specifically, elevated cytoplasmic LMTK3 levels were strongly associated with a higher risk of mortality and poorer survival, particularly during the first few years post-diagnosis. Furthermore, the introduction of LMTK3-binding peptides (LMTK3BPs), which exhibit high specificity and efficacy against both chemosensitive and chemoresistant OC cells, represents a promising new opportunity for targeted treatment strategies (Figure 1).

**Figure 1:**
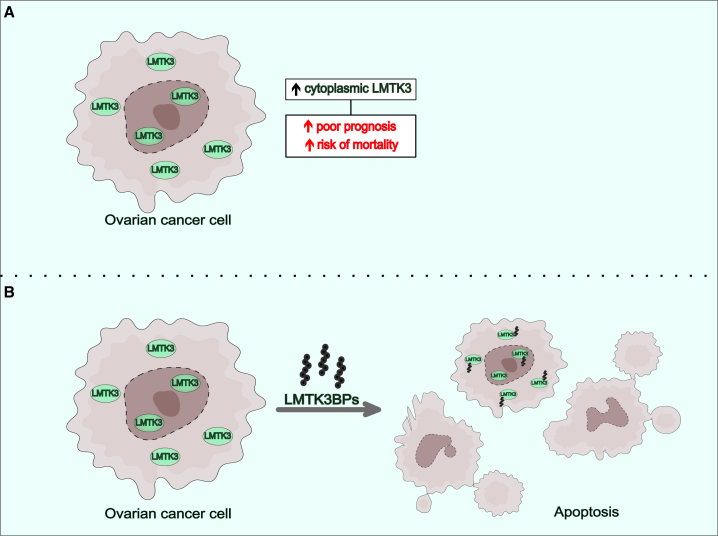
LMTK3 as a biomarker and therapeutic target using LMTK3BPs in early-stage OC (A) In ovarian cancer (OC) cells, LMTK3 is localized in both the nucleus and cytoplasm. Higher cytoplasmic LMTK3 levels are associated with increased poor prognosis and increased risk of mortality. (B) Treatment of OC cells with LMTK3BPs induces apoptosis, demonstrating their potential as a targeted therapeutic strategy.

OC is marked by poor survival rates, primarily due to late diagnosis and the frequent emergence of chemotherapy resistance.^2^ The disease is often detected at an advanced stage because of the lack of reliable early detection biomarkers, and it has a high recurrence rate even after standard treatments, which typically include surgery and chemotherapy. As the molecular mechanisms driving OC are progressively uncovered, research has shifted toward identifying biomarkers that can predict patient prognosis and serve as potential therapeutic targets.

In this regard, LMTK3 has gained attention during the last decade due to its involvement in critical cellular processes such as cancer cell proliferation, invasion, migration and survival.^3^ While its role in OC is not fully understood, LMTK3 is overexpressed in several other malignancies, including breast, lung, gastric, colorectal, and blood cancer, while it may also act as a predictive and prognostic biomarker in certain tumor types.^4^ The present study is the first to assess LMTK3’s prognostic value in patients with early-stage OC and its therapeutic potential in OC.

LMTK3 was found to be abundant in both the nucleus and the cytoplasm of OC cells. Immunohistochemistry (IHC) analysis revealed that 67.5% of the tumors exhibited strong nuclear LMTK3 staining. Notably, nuclear LMTK3 was linked to better clinical outcomes, highlighting it as a favorable prognostic marker. In contrast, high levels of cytoplasmic LMTK3, especially when not accompanied by strong nuclear staining, were associated with an increased risk of death. This differential localization of LMTK3 offers valuable insights into its role in OC, suggesting that its presence at distinctive intracellular compartments, along with fluctuations of its expression levels, can have variable effects on tumor progression by regulating different cell signaling pathways.

Beyond its prognostic significance, the study also investigated LMTK3 as a promising therapeutic target in OC. The team identified specific LMTK3BPs and evaluated their effects in both *in vitro* and *in vivo* models. These peptides induced apoptosis in OC cells, including chemoresistant subtypes, without affecting normal ovarian cells. Notably, the cell-killing effects were enhanced when combined with standard chemotherapies such as cisplatin and docetaxel. In an orthotopic xenograft mouse model, treatment with LMTK3BPs resulted in a significant reduction in tumor size, with no signs of toxicity in normal tissues. From a therapeutic standpoint, the development of LMTK3BPs that specifically target cancer cells without damaging normal tissues is particularly promising, as it addresses a critical challenge in cancer treatment: maximizing efficacy while minimizing toxicity. Previously, small-molecule inhibitors targeting LMTK3 have shown similarly significant promise in cancer therapy. In a recent study, C28, a potent small-molecule inhibitor of LMTK3, was found to decrease proliferation in a panel of 60 human cancer cell lines including OC cells and induce apoptosis in breast cancer cells.^5^ Interestingly, C28 effectively inhibited tumor growth in both orthotopic and transgenic breast cancer mouse models while sparing normal epithelium. In addition to C28, another small-molecule LMTK3 inhibitor, C36, has been developed, demonstrating similar antiproliferative effects in various cancer cell lines.^6^ In the present study, the peptide-based approach of LMTK3BPs adds a new perspective to LMTK3-targeting strategies, offering an additional/alternative option to small-molecule inhibitors and expanding therapeutic choices in cancer treatment.

Moreover, the synergistic effects observed between LMTK3BPs and chemotherapy offer exciting possibilities for the application of combination therapy, particularly for patients with chemoresistant tumors. Given that patients with OC often relapse with drug-resistant disease, these peptides could provide a much-needed alternative or adjunct to existing treatments. Taking into consideration the overexpression of LMTK3 in various types of cancers, LMTK3BPs could ultimately be applicable to other solid tumors.

Despite the novel and promising results, several caveats require further exploration. The study primarily used early-stage OC samples, and ongoing research is needed to validate these findings in advanced-stage disease, where treatment resistance is more prevalent. Validation in additional cohorts encompassing a larger number of patients would further support the observations described in this study. Although the *in vivo* experiments demonstrated the safety and non-toxic effects of LMTK3BPs, a major barrier to clinical translation lies in achieving efficient intracellular delivery of these peptides to cancer cells. This challenge could possibly be addressed through alternative approaches such as gene therapy. Utilizing gene delivery methods—like oncolytic viruses or lipid nanoparticles (LNPs) carrying genes encoding LMTK3BPs—could ultimately enhance the peptides’ uptake and efficacy. Another important question that requires elucidation is the exact mechanism by which LMTK3 exerts its dual effects in the nucleus and cytoplasm. Understanding this mechanism could pave the way for even more targeted therapeutic strategies.

Overall, the study’s findings provide a solid foundation for the development of LMTK3-targeted therapies that could be used in combination with existing treatments to improve OC patient outcomes, especially in the context of chemoresistant disease. As research into LMTK3 continues, its role as a biomarker for predicting patient prognosis could also enhance personalized treatment strategies, allowing clinicians to tailor therapies based on LMTK3 expression levels. With small-molecule inhibitors already developed and the LMTK3BPs introduced in this study, both strategies could ultimately be explored as new complementary treatment options alongside existing therapies, not only in OC but also in a range of other cancers where LMTK3 is implicated.

## Declaration of interests

G.G. is the founder and chief scientific officer of Stingray Bio.
